# Statistical feature training improves fingerprint-matching accuracy in novices and professional fingerprint examiners

**DOI:** 10.1186/s41235-022-00413-6

**Published:** 2022-07-16

**Authors:** Bethany Growns, Alice Towler, James D. Dunn, Jessica M. Salerno, N. J. Schweitzer, Itiel E. Dror

**Affiliations:** 1 College of Social Sciences and International Studies, University of Exeter, Exeter, UK; 2 School of Social and Behavioural Sciences, Arizona State University, Tempe, USA; 3 School of Psychology, University of New South Wales, Kensington, Australia; 4 University College London, London, UK

**Keywords:** Training, Forensic science, Perceptual expertise, Face matching, Fingerprint matching, Expertise

## Abstract

Forensic science practitioners compare visual evidence samples (e.g. fingerprints) and decide if they originate from the same person or different people (i.e. fingerprint ‘matching’). These tasks are perceptually and cognitively complex—even practising professionals can make errors—and what limited research exists suggests that existing professional training is ineffective. This paper presents three experiments that demonstrate the benefit of perceptual training derived from mathematical theories that suggest statistically rare features have diagnostic utility in visual comparison tasks. Across three studies (*N* = 551), we demonstrate that a brief module training participants to focus on statistically rare fingerprint features improves fingerprint-matching performance in both novices and experienced fingerprint examiners. These results have applied importance for improving the professional performance of practising fingerprint examiners, and even other domains where this technique may also be helpful (e.g. radiology or banknote security).

## Significance statement

Forensic science experts carry out ‘matching’ tasks in the criminal justice system to link or exclude suspects from crime scenes. Despite the importance of this high-stakes task, examiners do make errors that can contribute to wrongful convictions. Existing research has shown that some current training programmes in forensic science are ineffective at improving performance in forensic science disciplines. The current research found that training people to focus on rare fingerprint features during fingerprint-matching improves the performance of novices and practising fingerprint examiners. These findings have important implications for training new and existing fingerprint examiners, as reducing their errors will help avoid wrongful convictions within the justice system.

## Introduction

Experts have skills and knowledge that give them a considerable advantage over novices for tasks within their domain of expertise (Ericsson et al., 2018). For example, fingerprint examiners are more accurate than novices at determining whether two fingerprints originate from the same source (i.e. the same person or different people; Busey & Vanderkolk, 2005; Thompson & Tangen, 2014; Ulery et al., 2011), and radiologists are more accurate at distinguishing between normal and abnormal radiographs than novices, (Azevedo et al., 2007; Evans et al., 2013; Treviño et al., 2020; Wu et al., 2019). In high-stakes ‘real-world’ domains such as these where high accuracy is paramount, there is a need for training interventions that improve the effectiveness and efficiency of experts. Expertise in a domain typically takes years of experience and deliberate practice to develop (Ericsson et al., 2018). However, in some domains, short perceptual training interventions that teach people to focus on particularly useful visual cues have been able to fast-track the development of expertise (Dror et al., 2008; Towler et al., 2021).

One method of identifying useful visual cues to include in perceptual training is an ‘expert knowledge elicitation’ approach—where experts in a domain are studied to identify which cues they use so that those cues can then be taught to novices. An early example of this approach investigated the impact of perceptual training on a chicken sexing task (Biederman & Shiffrar, 1987)—a challenging task that requires fine discrimination of visual features. Researchers interviewed experienced professional chicken sexers with 18–36 years’ experience and discovered a single important diagnostic feature that indicated a chicken’s sex: males had a convex genital bead, whilst females had a concave or flat genital bead. Only one minute of brief training to utilize this visual cue was needed to increase novices’ ability to sex chicks increased by nearly 40%.

More recently, researchers have used this approach to investigate the expertise of forensic facial examiners who distinguish between photographs of the same person and different people (Towler et al., 2021). This is another challenging perceptual task—particularly for unfamiliar faces (Megreya & Burton, 2006)—and facial examiners are typically trained over many years of mentorship and experience (Towler et al., 2021). Researchers identified particularly diagnostic visual features in faces (e.g. ears, scars, and moles) that were predictive of examiners’ superior performance (Towler et al., 2017)—and subsequently trained novices to focus on these diagnostic features when matching faces (Towler et al., 2021). After only six minutes of training to focus on these features, novices’ face-matching accuracy increased by 6%—equivalent to approximately *half* of facial expert examiners’ superiority in this task—and more effective than many industry training courses that take much longer to complete (Towler et al., 2019).

An alternative method to eliciting useful visual cues to include in perceptual training for experts can be drawn from prominent mathematical theory. Information theory suggests that rarer features can provide a useful diagnostic cue for discrimination or categorization (Busey et al., 2016; Shannon, 1948)—an approach also applicable to many other cognitive processes (e.g. attention and visual search; Bruce & Tsotsos, 2009; or perceptual learning; Gibson, 1969). For example, two fingerprints that share a rare fingerprint feature (e.g. a ‘lake’) would be more likely to come from the same person, than two fingerprints that share a common feature (e.g. a ‘bifurcation’; see also Gutierrez-Redomero et al. (2011), Gutiérrez-Redomero et al. (2012 for fingerprint minutiae frequencies). In another study, researchers trained novices to focus on statistically rare features across a set of artificial patterns when deciding if the two patterns were the same or different (Growns & Martire, 2020a). After less than two minutes of statistical feature training, novices’ accuracy in this task improved by 13%, achieving better performance than untrained novices and forensic science examiners who complete similar comparison tasks professionally.

These studies demonstrate that perceptual training can improve performance in visual decision-making tasks—specifically the importance of utilizing particular visual cues that are diagnostic in a domain. Yet no research has focused on the potential importance of statistically derived training in real-world decision-making (e.g. fingerprint or face-matching). This is important as the expert-elicitation approach for developing training may not be possible in all domains—particularly when experts are not explicitly aware of the processes underlying their decision-making (Ericsson et al., 2018). For this reason, perceptual training that exploits quantifiable statistical information offers a viable alternative pathway for developing programs that fast-track the development of expertise—especially in domains where existing training is ineffective (e.g. forensic science; Towler et al., 2019).

In this paper, we present three experiments that examine the benefit of statistical feature training on a visual comparison task with important applied implications: fingerprint comparison. We present two experiments that investigate the impact of statistical feature training on novices (Exps. 1–2) and professional fingerprint examiners (Exp. 3)—as limited research has explored the potential for perceptual training to improve expert decision-making. Although experts outperform novices in tasks within their domain of expertise, they do still make errors. For example, even professional fingerprint examiners have error rates ranging from 8.8 to 35% in fingerprint comparison tasks—depending on task difficulty (Busey & Vanderkolk, 2005; Ulery et al., 2011). Therefore, there is still room to improve expert performance to further reduce mistakes that are made—particularly in real-world domains like forensic science where errors can result in life-altering consequences, such as wrongful convictions.

## Experiment 1

Experiment 1 examined the impact of statistical feature training on novices’ fingerprint comparison performance. We also included face comparison as a baseline control task. We adapted the statistical feature training module from Growns and Martire (2020a) to include examples of statistically rare and common features in fingerprints and faces. We compared the impact of training on novices’ visual comparison performance by comparing the change between trained novices’ performance pre-to-post-training to untrained novices’ change in performance. We investigated fingerprint and face comparison in the current study as there is quantified statistical data available on the frequency of features in these domains, and the bulk of the research in forensic expertise has been conducted in fingerprint and face comparison (see Growns and Martire (2020b) for review).

### Method

#### Design

We used a 2 between-subjects (training: statistical feature or control) × 2 within-subjects (time: pre-training or  post-statistical feature training) mixed design. The pre-registration, data, and analysis scripts can be found at https://osf.io/jpxwe/.

#### Participants

We recruited 143 participants online via Prolific Academic based on an a priori power analysis for detecting a medium effect (*f* = 0.25) in our design with 80% power (including an additional 10% to account for attrition) using the *WebPower* package in *R* (Zhang & Yuan, 2018). This effect size was chosen as previous studies examining the impact of similar training on visual comparison performance have identified medium effects (e.g. Growns & Martire, 2020a; Towler et al., 2017). To be eligible for the study, participants were required to have normal or corrected-to-normal vision, to live in the USA, to have a Prolific approval rating of 95% + , and to have completed the experiment on a tablet or computer (not a cellular device). Participants were excluded if they failed at least three (out of five) attention-check questions (*n* = 44).^1^

Participants in the final sample (*n* = 99) were 32.4 years (*SD* = 11.1, *range* = 18–68), and about half (52.5%) self-identified as male (45.4% as female and 2% as gender diverse). Each participant was compensated US$5.20 for completing the approximately 50-min experiment.

### Materials

#### Comparison tasks

Participants completed face and fingerprint comparison tasks both before and after training and completed each pre-training and post-training task with different trials.

**Fig. 1:**
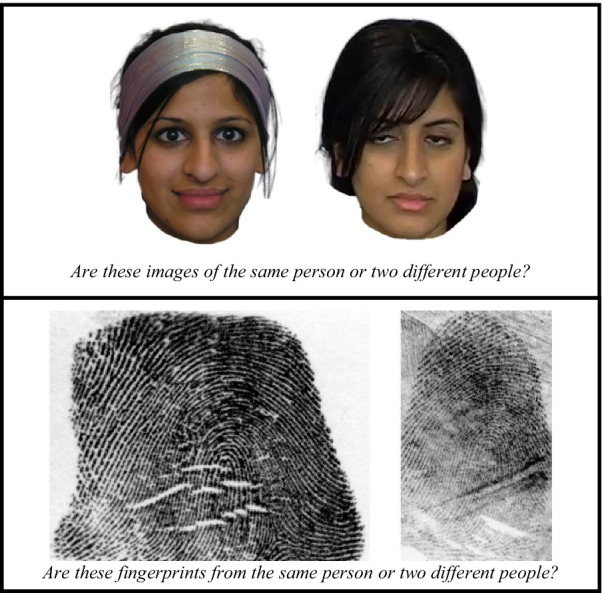
Example ‘match’ trials for both matching tasks (face: upper panel; fingerprint: lower panel)

*Face comparison* Participants completed a standardized test of face comparison as a baseline control task: the Glasgow Face-Matching Task-2 (GFMT2-SA and SB; White et al., 2021, p. 2; see upper panel of Fig. 1) where participants view two faces side by side and were asked ‘are these images of the same person or two different people?’ on each trial. They responded by selecting one of two buttons (‘same’ or ‘different’) at the bottom of the screen. Participants completed 80 face comparison trials in total: 40 trials pre-training and 40 trials post-training in a randomized order. Participants completed 40 different trials (20 match and 20 non-match different trials at each time period) pre-training and post-training.

The GFMT2 was designed to be a challenging and representative task of face comparison accuracy by calculating item-to-test correlations for each trial, and the 40 match and 40 non-match trials with the highest correlations were then selected and divided into two equally difficult forms of the test.

*Fingerprint comparison* Participants completed a standardized test of fingerprint comparison: we developed this test using the same psychometric method used to develop the GFMT2 (see below for more detail; adapting trials from Growns and Kukucka (2021); see lower panel of Fig. 1). On each trial, participants viewed two fingerprints side by side and were asked ‘are these fingerprints from the same person or two different people?’ on each trial. They responded by selecting one of two buttons (‘same’ or ‘different’) at the bottom of the screen. Participants completed 80 fingerprint comparison trials in total: 40 trials pre-training and 40 trials post-training (20 match and 20 non-match at each time period) in a randomized order. Participants completed 40 different trials (20 match and 20 non-match different trials at each time period) pre-training and post-training.

Fingerprint comparison trials were drawn from a database of over 1,000 fingerprints that were recorded by a qualified fingerprint examiner (see Growns & Kukucka, 2021 for additional detail). Fingerprints in this database were clear, rolled exemplar fingerprints and latent fingerprints collected from a variety of different surfaces (e.g. plastic or glass) and developing techniques (e.g. aluminium, black, or magneto flake powder). Each trial consisted of one exemplar and one latent fingerprint: match trials consisted of one exemplar and one latent fingerprint from the same individual, and non-match trials consisted of one latent fingerprint and one similar exemplar fingerprint identified via an Automated Fingerprint Identification System (AFIS; Dror & Mnookin, 2010).

Trials were selected using the same method used to create the GFMT2—we calculated item-to-test correlations for each trial (i.e. how well accuracy on each trial predicts each participants’ overall performance) using pilot data from Growns and Kukucka (2021). We then selected the 80 trials (40 match and 40 non-match) that had the highest item-to-test correlations and then divided the trials into two equally difficult versions of the test (comprising 20 match and 20 non-match trials each). We used this method as it identifies trials that are most predictive of overall test performance and provides an overall estimate of a trial’s contribution to test reliability (Guilford, 1954; see also White et al, 2021 and Wilmer et al., 2012).

**Fig. 2:**
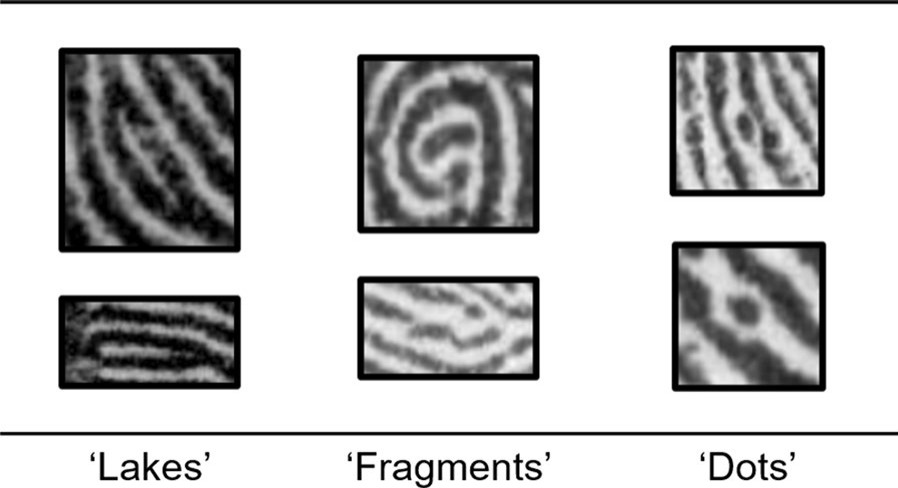
Examples of rare minutiae in the fingerprint comparison task

**Fig. 3:**
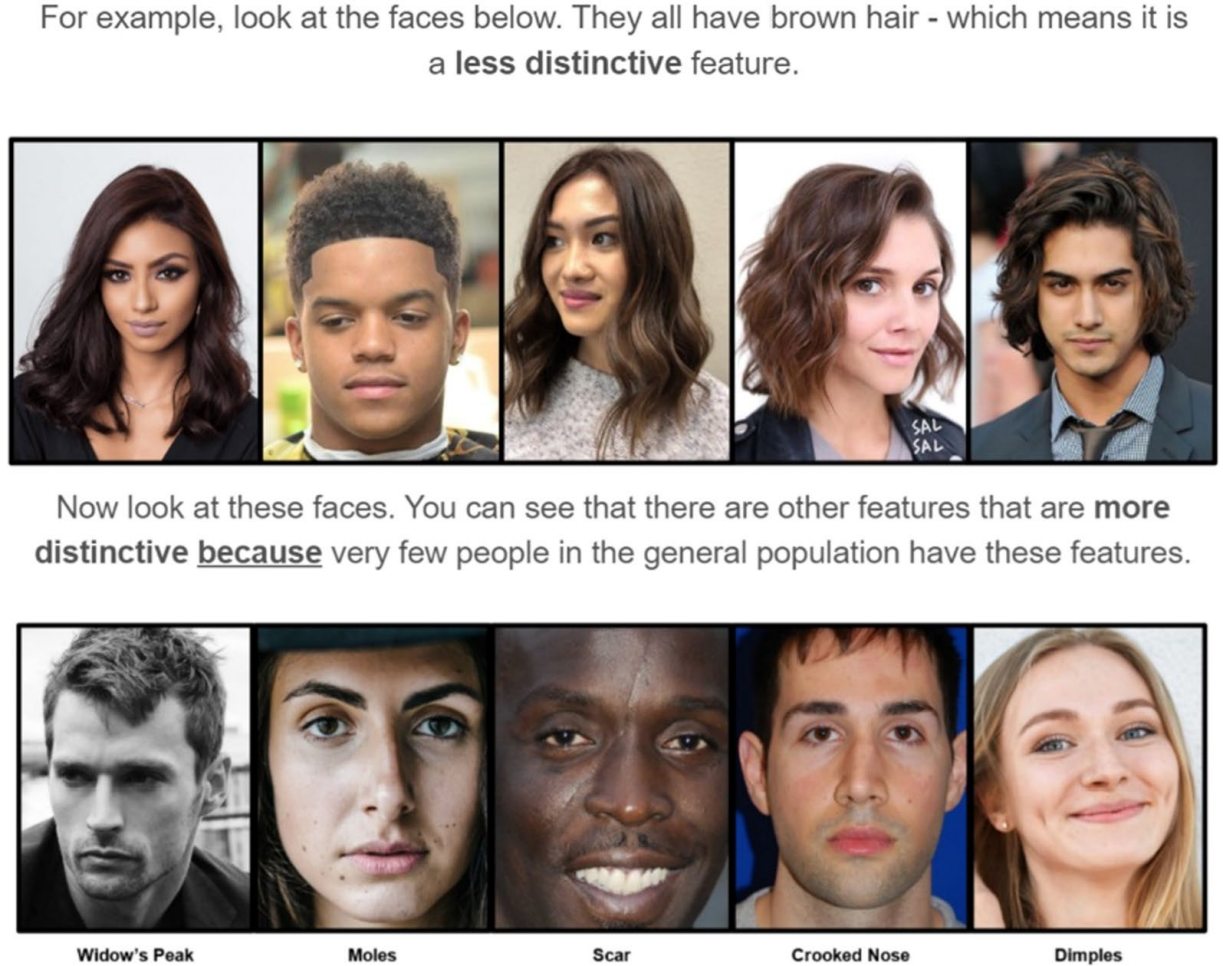
Examples of diagnostic and less diagnostic facial features shown to participants in the statistical feature training module

It is also important to note that we did not deliberately select trials that contained rare minutiae as it was not feasible for the examiner who collected the stimuli to identify all minutiae in each fingerprint (e.g. a single fingerprint can contain between 40 and 100 minutiae; Zaeri, 2011). We were thus unable to calculate the total proportion of all rare and common minutiae contained in the fingerprint participants viewed. We instead elected to select trials that were most predictive of performance (as described above). Nevertheless, the fingerprint trials used in the present study did contain rare minutiae—for example, the ‘lakes’, ‘fragments’, and ‘dots’ that can be seen in Fig. 2 (see also Fig. 3 for additional examples).

#### Training module

Participants were randomly assigned to either complete the statistical feature training module or the control training module.

##### Statistical feature training

Participants completed an adaption of the statistical feature training from Growns and Martire (2020a, 2020b; see Supplementary Analyses on OSF for full transcript) where participants were trained to use statistically rare and common features in faces and fingerprints. The training was adapted to include real-world examples of statistical features in faces and fingerprints and took approximately five and a half minutes to complete (*M* = 339 s, SD = 7 s).

Participants were first asked to imagine they were a police officer needing to compare photographs of people (Section 1 of the training). They viewed two hypothetical cases: one where two photographs shared a statistically rare feature (i.e. a large scar; Case 1), and one where two photographs shared a statistically common feature (i.e. brunette hair; Case 2). They were asked which case was more likely to show the same person (Case 1 or 2) and were provided with corrective feedback (Case 1 was correct).

In Section 2 of the training, they were then informed that statistically rare features helped in comparison tasks and were instructed to use similar rare features in faces in their decisions, rather than common features (e.g. brunette hair). They were shown visual examples of rare and common features in individual faces (although we used the term ‘distinctive’ rather than ‘diagnostic’ to reduce jargon in the experiment; see Fig. 3). Statistically rare (e.g. moles, scars, crooked noses, dimples, or widow’s peaks) and common (e.g. brunette hair) features in faces were chosen.

**Fig. 4:**
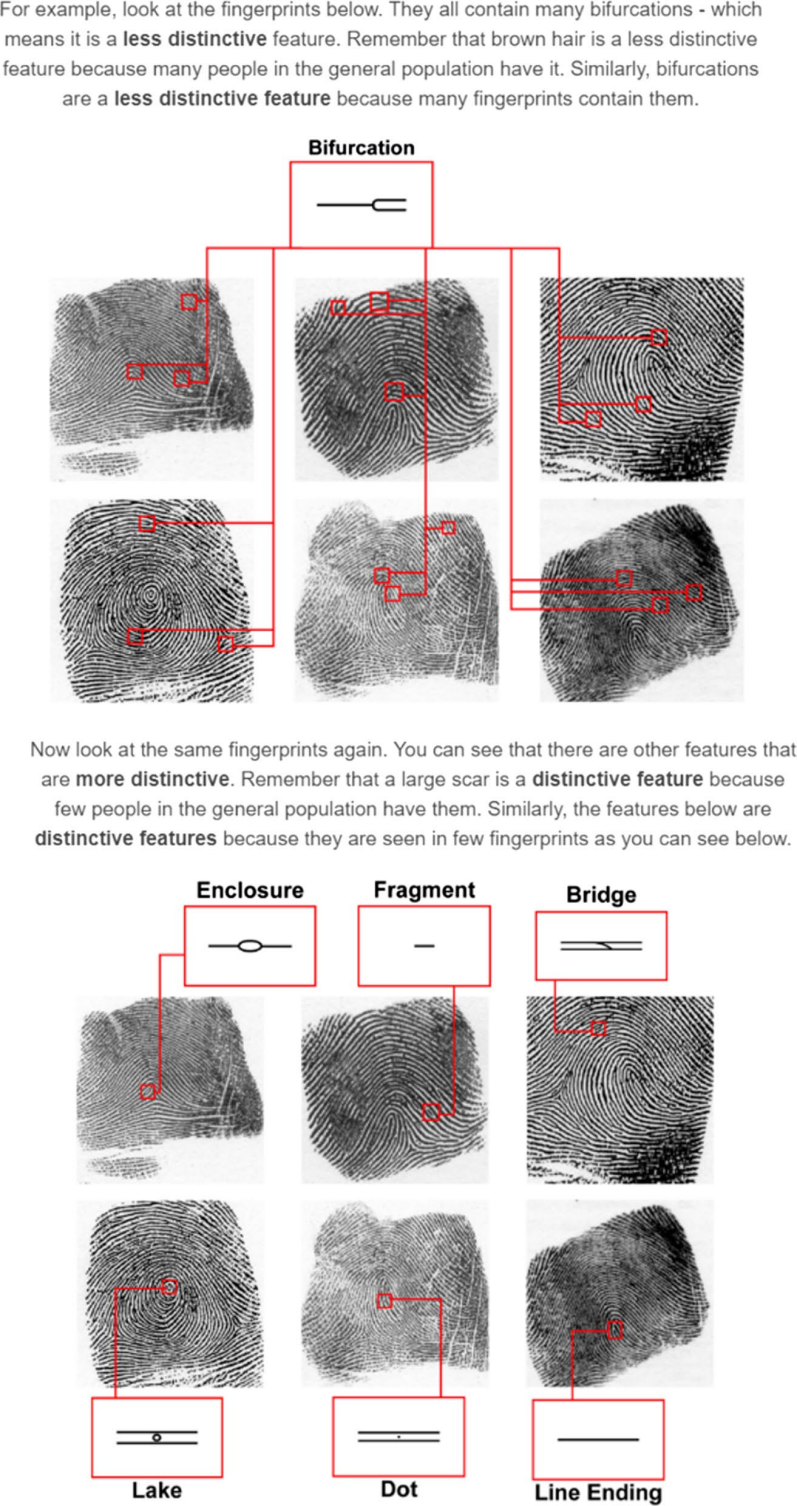
Examples of diagnostic and less diagnostic fingerprint features shown to participants in the statistical feature training module. Note the size of these images has been scaled for the manuscript and the images participants viewed were larger

In Section 3 of the training, participants were then informed a similar theory applied for fingerprint comparison and were shown visual examples of different fingerprint features (e.g. bifurcations, enclosures, or dots; see Fig. 4). They were instructed to look for and use statistically rare features in fingerprints in their decisions, rather than common features. They were then shown visual examples of rare and common features in individual fingerprints (see Fig. 4). Rare (e.g. enclosures or dots) and common features (e.g. bifurcations) in fingerprints were chosen (see Gutierrez-Redomero et al., 2011; Gutiérrez-Redomero et al., 2012 for fingerprint minutiae frequency data).

##### Control training

Participants completed a brief conflict resolution course as a control training module adapted from Towler et al. (2021). Participants were informed about different styles of conflict and strategies for conflict resolution. The control training module took approximately four minutes to complete (*M* = 244 s, SD = 18 s).

### Procedure

Participants completed the experiment via the online survey platform Qualtrics (2005). Participants were randomly assigned to training conditions (statistical feature or control) and then completed the pre-training face and fingerprint comparison tasks in a randomized order. Participants completed one set of trials in the pre-training phase of the experiment and then completed a different set of trials in the post-training phase. All participants in both conditions completed the same set of trials in each phase to minimize any potential error variance that could be introduced by participants completing different trials during different stages of the experiment (see Mollon et al., 2017 for discussion, and note our analyses control for trial-level variance—see Appendix).

At the beginning of each comparison task, participants received brief task instructions and completed two practice trials where they were given corrective feedback (one match and one non-match). Participants in the statistical feature training condition then completed the training module (henceforth *trained novices*), whilst those in the control training condition completed the conflict resolution module (henceforth *untrained novices*). Thereafter, all participants completed the post-training face and fingerprint comparison tasks in a randomized order between participants. Upon completion of the comparison tasks, participants provided demographic information and then viewed a debriefing statement.

#### Dependent measures

Comparison performance in each task was assessed via signal-detection measures of sensitivity (d') and bias (C) (Phillips et al., 2001; Stanislaw & Todorov, 1999). Higher d' values indicate higher sensitivity to the presence of a target stimulus, and higher values are typically interpreted as higher ‘accuracy’ in a task. Positive C values indicate an increased tendency to judge stimuli pairs as a ‘non-match’, whilst negative C values indicate an increased tendency to judge stimuli pairs as a ‘match’. We also pre-registered analyses examining raw accuracy which are reported in Table 1 in Appendix.

## Results

**Fig. 5:**
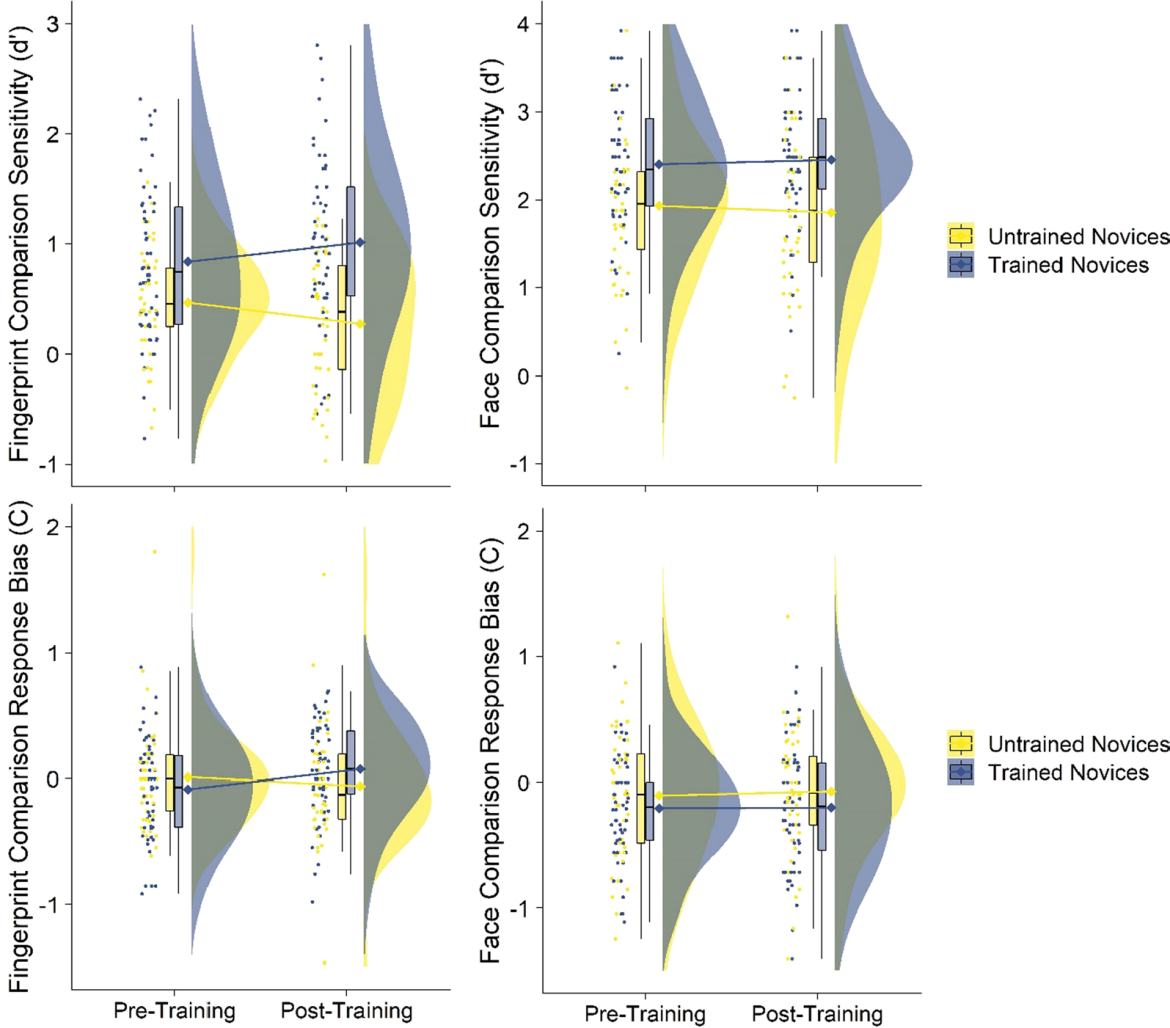
Sensitivity (upper panel) and response bias (lower panel) in the fingerprint (left panel) and face (right panel) comparison tasks by time and condition in Experiment 1. Raincloud plots depict (left-to-right) the jittered participants’ averaged data points, box-and-whisker plots, means (represented by diamonds) with error bars representing ± 1 SE, and frequency distributions

We conducted logistic mixed-effects regression models to explore fingerprint and face comparison using the *lme4* and *lmerTest* packages in R (Bates et al., 2014, p. 4; Kuznetsova et al., 2017), with the *emmeans* package used to explore any follow-up comparisons (Russell, 2018). We predicted sensitivity and response bias from the interaction between time (pre-training or post-training) and condition (trained novices who received statistical feature training or untrained novices in the control condition who received the conflict resolution training), with a random effect included for participant (see Fig. 5).

### Fingerprint comparison performance

#### Sensitivity

Trained novices’ (*M* = 0.92, SD = 0.75) finger comparison sensitivity was significantly higher than untrained novices (*M* = 0.37, SD = 0.56; *b* = 0.37, *t*_(140.80)_ = 2.71, *p* = 0.008, 95% CI[0.10, 0.63]), and there was also a small significant increase in all participants’ sensitivity pre-to-post-training (pre: *M* = 0.67, SD = 0.64, post: *M* = 0.68, SD = 0.80; *b* = − 0.19, *t*_(97)_ = 2.22, *p* = 0.029, 95% CI[− 0.37, − 0.02]). The interaction of interest between time and condition was also significant (*b* = 0.37, *t*_(97)_ = 3.13, *p* = 0.002, 95% CI[0.14, 0.60]). Trained novices’ sensitivity significantly increased pre-to-post-training (pre: *M* = 0.84, SD 0.71, post: *M* = 1.01, SD = 0.79; *t*_(141)_ = 5.46, *p* < 0.001), whilst untrained novices significantly decreased (pre: *M* = 0.47, SD = 0.50, post: *M* = 0.28, SD = 0.61; *t*_(141)_ = 2.71, *p* = 0.008).

#### Response bias

The interaction of interest between time and condition for fingerprint comparison response bias was significant (*b* = 0.23, *t*_(97)_ = 3.15, *p* = 0.002, 95% CI[0.09, 0.37]). Trained novices’ response bias significantly shifted positively pre-to-post-training (pre: *M* = − 0.08, S*D* = 0.40, post: *M* = 0.08, SD = 0.38; *t*_(97)_ = 3.24, *p* = 0.002), indicating a greater likelihood to say the fingerprints were from different people after training, whilst untrained novices’ bias did not significantly differ pre-to-post-training (pre: *M* = 0.01, SD = 0.42, post: *M* = − 0.06, SD = 0.46; *t*_(97)_ = 1.31, *p* = 0.195). The main effects of time (*b* = − 0.07, *t*_(97)_ = 1.31, *p* = 0.195, 95% CI[− 0.17, 0.04]) and condition (*b* = − 0.10, *t*_(97)_ = 1.40, *p* = 0.256, 95% CI[− 0.26, 0.07]) were not significant.

### Face comparison performance

#### Sensitivity

Trained novices’ (*M* = 2.43, SD = 0.73) face comparison sensitivity was significantly higher than untrained novices (*M* = 1.89, SD = 0.89; *b* = 0.47, *t*_(152.69)_ = 2.90, *p* = 0.004, 95% CI[1.69, 2.17]). However, the main effect of time was not significant (*b* = -0.08, *t*_(97)_ = 0.65, *p* = 0.520, 95% CI[− 0.31, 0.16]), nor was the interaction of interest between time and condition for face comparison sensitivity (*b* = 0.13, *t*_(97)_ = 0.79, *p* = 0.431, 95% CI[− 0.19, 0.44]). This indicates that training had no impact on participant’s face sensitivity pre-to-post-training.

#### Response bias

The main effects of time (*b* = − 0.10, *t*_(148.56)_ = 1.06, *p* = 0.293, 95% CI[− 0.10, 0.17]) and condition (*b* = 0.03, *t*_(97)_ = 0.50, *p* = 0.620, 95% CI[− 0.29, 0.04]) were not significant for face comparison response bias, nor was the interaction between time and condition (*b* = − 0.03, *t*_(97)_ = 0.31, *p* = 0.756, 95% CI[− 0.21, 0.15]). This indicates that training had no impact on participant’s face response bias pre-to-post-training.

## Discussion

Experiment 1 examined whether statistical feature training improves novices’ face and fingerprint comparison performance. Whilst training was ineffective in improving face comparison performance, it did improve fingerprint comparison performance. Trained novices’ fingerprint comparison performance increased pre-to-post-training compared to untrained novices overall—whose performance actually decreased pre-to-post-training. This decrease in untrained novices’ performance may be due to possibly distracting participants by asking them to focus on irrelevant information (further investigated in Experiment 2). Nevertheless, trained novices’ performance did increase—a performance boost that was largely driven by accuracy in non-match trials (see Table 1 and Fig. 10 in Appendix) and an increased conservatism in their tendency to respond ‘non-match’. Given that the statistical feature training module took only five and a half minutes to complete, this suggests that this type of training could be a fast and effective way to boost performance in new fingerprint trainees—particularly on the type of comparison that can result in the wrongful conviction of innocent people (i.e. non-match errors).

Statistical feature training did not improve novices’ face comparison accuracy—on either match or non-match trials. This is in contrast to the success of statistical feature training for fingerprint comparison and to previous research showing that face comparison is improved by focusing on similar diagnostic features derived via expert-elicitation methods (Towler et al., 2021). There is some overlap between the diagnostic features used in the current statistical feature training (e.g. facial marks and scars are featured in statistical feature training in Towler et al. (2021)), but also some differences (e.g. ears are not in statistical feature training, but are in Towler et al., 2021). Different features may be useful in different visual comparison tasks. For example, visual cues elicited via expert-elicitation methods might be more useful in familiar visual tasks (i.e. faces), whilst statistically derived methods are more useful in unfamiliar visual tasks (i.e. fingerprints).

To explore this possibility, we conducted a pilot experiment where we added a single slide to the training module instructing participants to specifically pay attention to the expert-derived diagnostic features from Towler et al. (2021): ears and facial marks (i.e. scars, freckles, and blemishes). Importantly, this training module improved both face and fingerprint comparison performance (see Pilot Study on OSF for full details). Therefore, it is important to ensure that training modules designed to improve visual comparison performance include the appropriate visual cues that will assist decision-making.

In Experiment 2 , we investigate whether the training effects observed in Experiment 1 are the result of domain-specific (i.e. fingerprint-specific training improving fingerprint comparison) or domain-combined (i.e. face and fingerprint-specific information). As expertise is typically regarded as narrow and domain-specific (Chase & Simon, 1973; Ericsson et al., 2018) and rarely generalizes beyond an expert’s domain of experience, we sought to investigate whether novices could benefit from only domain-specific training, or whether domain-specific (i.e. fingerprint) and domain-general (i.e. face) combined information is needed to improve performance. To do so, we compared the effect of domain-specific training alone versus domain-combined training alone on pre-to-post-performance, compared to control.

## Experiment 2

Experiment 2 examined whether the benefit of statistical feature training modules on novices’ fingerprint comparison performance is contingent on the combination of both domain-specific and domain-general statistical feature information. To assess this, participants were either given domain-combined statistical feature training (i.e. face *and* fingerprint information combined; henceforth *domain-combined trained novices*), domain-specific statistical feature training (i.e. fingerprint information only; henceforth *domain-specific trained novices*) or control training (i.e. untrained novices who completed the conflict resolution module from Experiment 1).

### Design

We used a 3 between (training: absent, domain-combined or domain-specific) × 2 within-subjects (time: pre-training or post-training) design. The pre-registration (including an update to our pre-registration to denote the collection of the additional data, data, and analysis scripts can be found at https://osf.io/jpxwe/.

### Participants

We recruited 348 participants online via Prolific Academic based on two a priori power analyses as in Experiment 1 for detecting medium effects (*f* = 0.25) in the study design with 80% power, including an additional 10% to account for attrition (see below for further discussion of data collected at two time periods). Participants were required to meet the same selection criteria as in Experiment 1 to be eligible for the study and were not eligible to participate if they had completed Experiment 1. Participants were excluded if they did not correctly pass at least two (out of three) attention-check questions (*n* = 7).

Participants in the final sample (*n* = 348) were 32.7 years old on average (SD = 14.0, *range* = 18–73), and the majority (65.80%) self-identified as female (33.05% male; 1.15% gender diverse). Each participant was compensated US$3.25 for completing the approximately 25-min experiment.

### Materials and analyses

Participants completed the experiment via the online survey platform Qualtrics (2005). Participants completed the same pre-training and post-training tasks (*n* = 40 trials per task) from Experiment 1. Participants in the control condition completed the same conflict resolution module from Experiment 1, and participants in the domain-combined training condition completed the entire module from Experiment 1. Participants in the domain-specific condition completed an adapted version containing only Sections 1 (i.e. the introduction portion) and 3 (i.e. the domain-specific portion) from the module from Experiment 1.

We collected data over two time periods: the first data collection contained participants from the domain-combined and domain-specific training conditions only, and the second data collection contained participants from all three conditions (see Pre-Registration on OSF). We collected the additional data from untrained novices in the second data collection period to ensure we had an appropriate control condition in Experiment 2 and collected additional data in both training conditions at the same time so that time period and condition were not confounded. We collected participants based on two separate power analyses for detecting medium effects in each study design at each time point (*n* = 141 in the 2 × 2 design and *n* = 174 in the 3 × 2 design in first and second data collection periods, respectively, plus 10% for data attrition in each experiment).

To simplify analyses, we pooled the data from the two time periods for time periods for analysis and conducted further analyses to control for any potential impact of sample collected during the first and second data collection periods on the results. As sample was not significant in any of these analyses and the pattern of results was consistent between this analysis and the pooled analysis (see Supplementary Analyses on OSF), we reported the pooled analyses in-text.

We also collected exploratory data in the second data collection period to examine whether participants reported using the statistical feature strategy during the post-training task. The majority of participants in both training conditions found the statistical feature strategy helpful (domain-specific 87.10%, domain-combined: 88.71%, whilst the majority in the control condition reported that conflict resolution training was not helpful (55.56%; see Supplementary Analyses on OSF).

### Procedure

Participants were randomly assigned to training conditions (control, domain-specific, or domain-combined), received brief instructions, and then completed the pre-training fingerprint comparison task including the two practice trials from Experiment 1. Participants then completed the training module relevant to their condition and subsequently the post-training fingerprint comparison task. Thereafter, all participants completed the post-training fingerprint comparison task, provided demographic information, and then viewed a debriefing statement.

## Results and discussion

### Fingerprint comparison performance

**Fig. 6:**
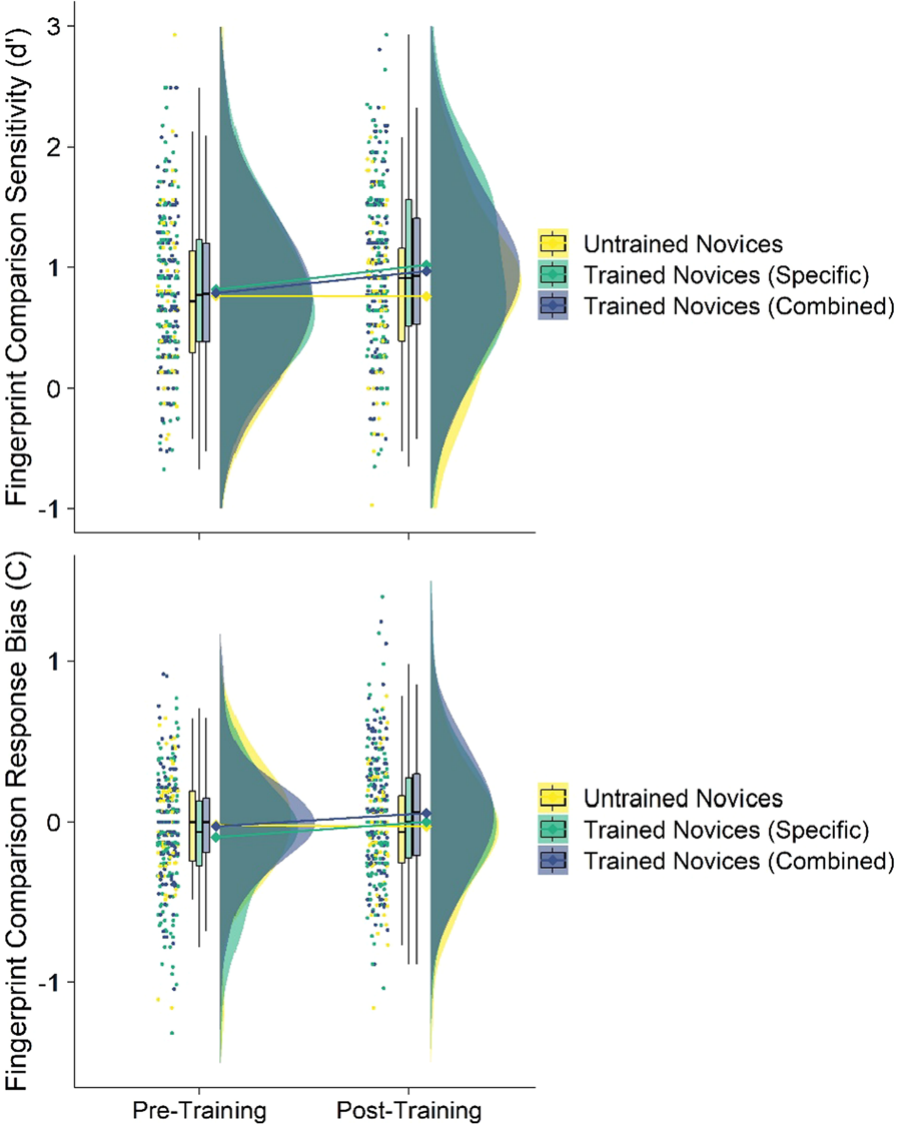
Sensitivity (upper panel) and response bias (lower panel) in the fingerprint comparison task by time and condition in Experiment 2. Raincloud plots depict (left-to-right) the jittered participants’ averaged data points, box-and-whisker plots, means (represented by diamonds) with error bars representing ± 1 SE, and frequency distributions

We conducted linear mixed-effect models on fingerprint comparison sensitivity and response bias from the interaction between time (pre-training or post-training) and condition (untrained novices, domain-combined trained novices, or domain-specific training novices), with a random effect included for participant (see Fig. 6). We also conducted analyses with sample included as a fixed effect and it was not significant in either analysis, and the pattern of results in this analysis was consistent with those reported in text (see Supplementary Analyses on OSF). We also pre-registered analyses examining raw accuracy which are reported in Table 2 in Appendix.

#### Sensitivity

The interaction between time and condition (untrained and domain-specific trained novices) was significant (*b* = 0.21, *t*_(344)_ = 2.13, *p* = 0.034, 95% CI[0.02, 0.40]) such that domain-specific trained novices significantly improved pre-to-post-training (pre: *M* = 0.80, S*D* = 0.62; *b* = − 0.20, *t*_(344)_ = 3.73, *p* < 0.001), but untrained novices did not (pre: *M* = 0.76, SD = 0.63, post: *M* = 0.75, SD = 0.72; *b* = 0.01, *t*_(344)_ = 0.07, *p* = 0.943). The interaction between time and condition (untrained and domain-combined trained novices) was also significant (*b* = 0.20, *t*_(344)_ = 2.01, *p* = 0.046, 95% CI[0.01, 0.39]) such that domain-specific trained novices significantly improved pre-to-post-training (pre: *M* = 0.78, SD = 0.62; *b* = − 0.19, *t*_(344)_ = 3.51, *p* = 0.001), compared to untrained novices.

The main effects of time (*b* = 0.01, *t*_(344)_ = 0.07, *p* = 0.943, 95% CI[− 0.17, 0.22]) and condition were not significant (post-domain-specific: *b* = 0.04, *t*_(541.97)_ = 0.42, *p* = 0.672, 95% CI[− 0.15, 0.24]; post-domain-combined: *b* = 0.02, *t*_(541.97)_ = 0.21, *p* = 0.836, 95% CI[− 0.17, 0.22]).

#### Response bias

The main effects of time (*b* < − 0.01, *t*_(344)_ = 0.04, *p* = 0.967, 95% CI[− 0.09, 0.09]) and condition were not significant (post-domain-specific: *b* = − 0.07, *t*_(541.73)_ = 1.30, *p* = 0.193, 95% CI[− 0.12, 0.07]; post-domain-combined: *b* < 0.01, *t*_(541.73)_ = 0.02, *p* = 0.985, 95% CI[− 0.19, 0.04]). The interactions between time and condition were also not significant (post-domain-specific: *b* = 0.10, t_(344)_ = 1.72, *p* = 0.086, 95% CI[− 0.01, 0.20]; post-domain-combined: *b* = 0.08, t_(344)_ = 1.44, *p *= 0.152, 95% CI[− 0.03, 0.19]).

These results are consistent with Experiment 1: domain-combined training improves novices’ fingerprint comparison sensitivity—an effect that is due to an increase in accuracy on non-match trials only (see Table 2 and Fig. 11 in Appendix). Experiment 2 also extended these results to reveal that domain-specific (i.e. fingerprint only) information is sufficient to also increase sensitivity via an improvement on non-match accuracy trials. It is important to note that we also did not see any decrease in our control condition in Experiment 2—indicating that the decrease seen in Experiment 1 may be spurious or due to the pre-existing differences between groups seen in this experiment. In sum, it is likely that conflict resolution training does not decrease fingerprint comparison performance.

In Experiment 3, we investigate whether statistical feature training can also improve the performance of practising fingerprint examiners. This is important as there is limited research about effective perceptual training programs in professional domains. We further investigate the impact of training content on fingerprint comparison performance by comparing the impact of domain-specific (i.e. fingerprint only) and domain-general (i.e. face only) training between novices and fingerprint examiners to investigate whether domain-general information can generalize to increase performance. If statistical feature training does improve performance, we would observe examiners’ performance improving pre-to-post-training (either domain-specific or domain-general). Conversely, examiners may already possess and rely on statistical information to facilitate their work and thus we could also observe no improvement from pre-to-post-training.

## Experiment 3

Experiment 3 examined the benefit of statistical feature training on examiners' and novices’ fingerprint comparison performance. We were also interested in whether the domain-general (i.e. face only) or domain-specific (i.e. fingerprint only) training section of the statistical feature training module enhanced fingerprint-matching performance.

### Method

#### Design

**Fig. 7:**
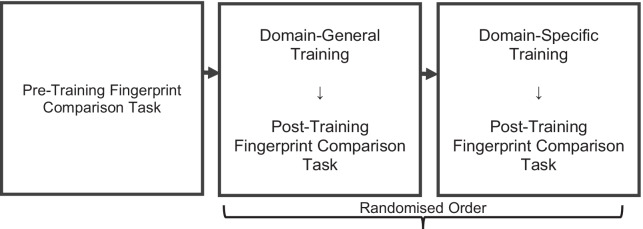
Procedure of tasks in Experiment 3

We used a 2 between-subjects (group: novices or examiners) × 3 within-subjects (time: pre-training, post-domain-specific training, or post-domain-general training) mixed design (see Fig. 7). The pre-registration, data, and analysis scripts can be found at https://osf.io/jpxwe/.

#### Participants

Fifty-two fingerprint examiners were recruited via a snowball-sampling method, and 52 novices were recruited from Prolific Academic. The sample size was determined by the number of fingerprint examiners recruited during our pre-registered time period for data acquisition and the subsequent sample-size-matched group of novices.

Initially, 95 participants were recruited through a snowball-sampling method via emails sent to forensic organizations and mailing lists. Based on our pre-registered criteria, all forensic practitioners who reported that fingerprint examination was not their primary area of training or specialization were excluded from the study (*n* = 40). These participants were excluded to ensure the homogeneity of the practitioner sample. Three additional participants were also excluded from the study as they reported having zero years’ experience (*n* = 2) or did not provide any information on their professional qualifications or practice to classify them as a fingerprint examiner (*n* = 1).

We then recruited the same number of novices (*n* = 52) via Prolific Academic who were required to meet the same criteria to qualify for the study as in Experiment 1.

Fingerprint examiners in the final sample (*n* = 52) were 43.3 years of age on average (SD = 9.16, range = 27–67) and about half reported they were male (53.9%; female = 44.2%, and gender diverse = 1.9%). Fingerprint examiners self-reported an average of 13.2 years professional experience (SD = 7.79, range = 1.5–37), having written an average of 1,147 court reports over the past ten years (SD = 1,858, range = 0–10,000), and the majority reported working for a police forensic laboratory (67.3%), 28.8% for a government forensic institution, 1.9% for a private forensic laboratory, and 1.9% for a university.

Novices in the final sample (*n* = 52) were 36.4 years of age on average (SD = 9.94, range = 21–69), and about half reported they were female (53.9%; 46.2% male). No participants from either sample failed our pre-registered attention-check criteria of not correctly answering three (out of four) attention-check questions.

Novices were paid $4.87 for participation in the approximately 45-min study, and examiners were not paid for their involvement. To motivate performance, all participants had the chance to win one of ten US$500 VISA gift cards that were awarded to the top ten performers across all tasks.

### Materials

#### Fingerprint comparison task

The fingerprint comparison trials from previous experiments were used, and we also added extra trials to create three fingerprint tasks for the pre-training, post-domain-general training, and post-domain-specific training phases with equal numbers of trials in each. To do so, we divided the trials from Experiment 1 (*n* = 80) and 10 additional trials (the 10 next highest item-to-test correlations from the Growns and Kukucka (2021) pilot data; Guilford (1954); see also White et al. (2021) and Wilmer et al. (2012) into three equally difficult tasks (*N* = 90; 30 trials per task). It is important to note that the rare minutiae may not have occurred in this task at the exact same frequencies as they do in the general population (e.g. Gutierrez-Redomero et al., 2011; Gutiérrez-Redomero et al., 2012). However, examiners may already have some underlying sense of these base rates as research demonstrates that fingerprint examiners can estimate the frequency of fingerprint stimuli better than novices (Growns et al., 2022; Mattijssen et al., 2020).

#### Training module

Participants in the domain-specific training condition completed the domain-specific training from Experiment 2 (i.e. the introduction Section 1 and the domain-specific Section 3 from Experiment 1). Participants in the domain-general training condition completed an adapted version of the training in Experiment 1 containing only the introduction Sections 1 and the domain-general Section 2.

### Procedure

All participants completed the experiment via Qualtrics (2005). They first provided brief demographic and professional practice information, received brief instructions, and then completed the pre-training fingerprint comparison task including the two practice trials from Experiment 1. Participants then completed two training modules, the domain-specific training (i.e. fingerprint-training) and the domain-general training (i.e. face-training), which were each proceeded by a post-training fingerprint comparison task. The order that these two training modules and the post-training fingerprint comparison task were completed was randomized. Finally, participants answered questions about their use of feature-comparison techniques in their work and were debriefed.

## Results and discussion

**Fig. 8:**
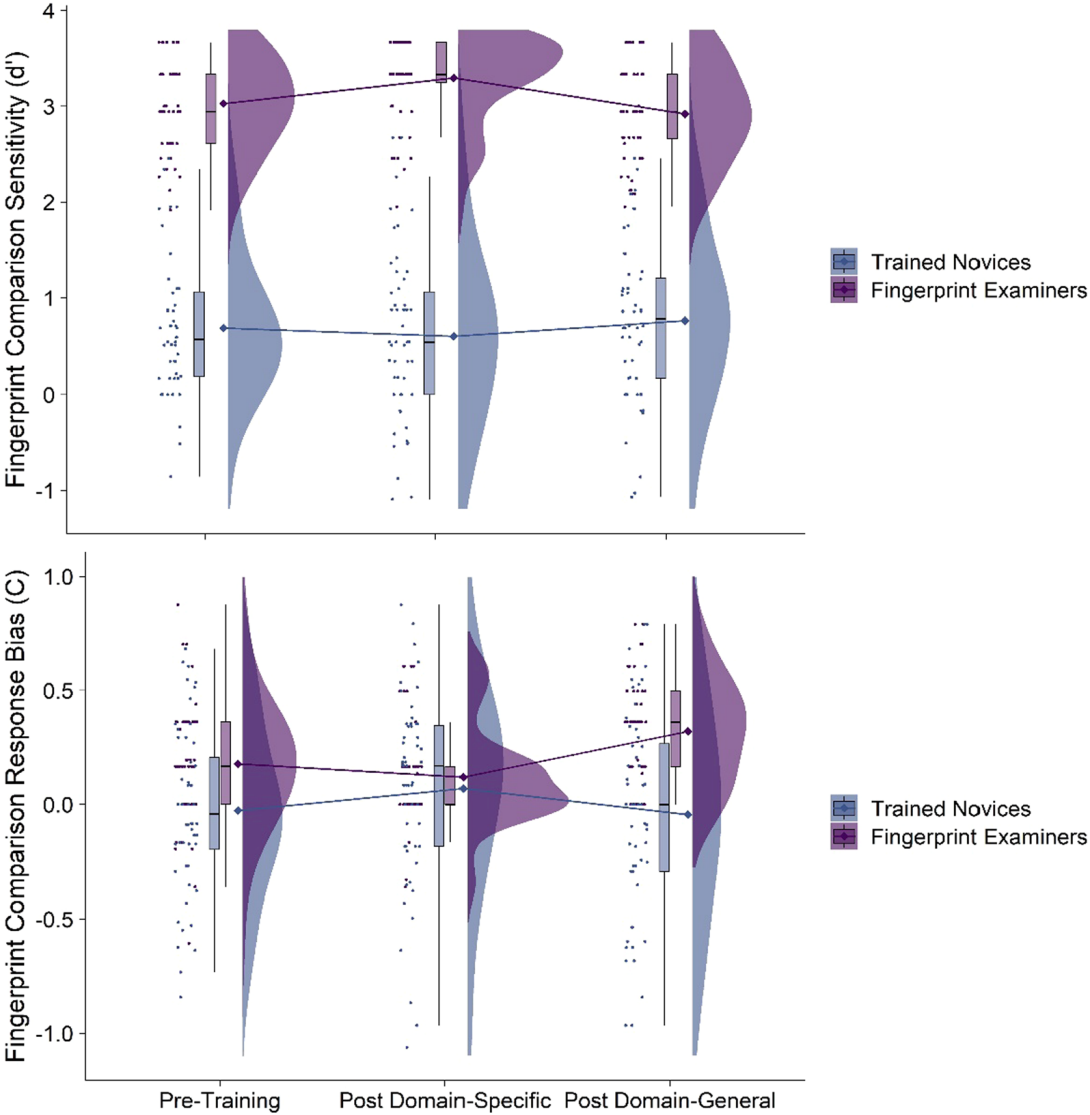
Sensitivity (upper panel) and response bias (lower panel) in the fingerprint comparison task by time and group in Experiment 3. Raincloud plots depict (left-to-right) the jittered participants’ averaged data points, box-and-whisker plots, means (represented by diamonds) with error bars representing ± 1 SE, and frequency distributions

We conducted linear mixed-effect models on fingerprint comparison sensitivity and bias from the interaction between time (post-domain-general training or post-domain-specific training, with pre-training as the reference category) and group (examiners or trained novices), with a random effect included for participant (see Fig. 8). As per our pre-registered analyses, we also conducted models with order of training (domain-specific or domain-general first) included as a fixed factor, but it was not significant in either model, and the pattern of results in this analysis was consistent with those reported in-text (see Supplementary Analyses on OSF). We also pre-registered analyses examining raw accuracy which are reported in Table 3 in Appendix. We also conducted exploratory analyses excluding trials with extreme values for any potential impact on our results but note that the pattern of results does not differ between these analyses (see Supplementary Analyses on OSF).

### Fingerprint comparison performance

*Sensitivity* Fingerprint examiners’ (*M* = 3.08, SD = 0.49) finger comparison sensitivity was significantly higher than trained novices (*M* = 0.68, SD = 0.80; *b* = 2.34, *t*_(172.17)_ = 18.16, *p* < 0.001, 95% CI[2.09, 2.69]), and the interaction between time (pre-training and post-domain-specific) and group was also significant (*b* = 0.35, *t*_(204)_ = 3.16, *p* = 0.002, 95% CI[0.13, 0.57]). Examiners’ sensitivity significantly increased pre-to-post after receiving domain-specific training (pre: *M* = 3.03, SD = 0.46, post: *M* = 3.30, SD = 0.45; *t*_(204)_ = 3.39, *p* = 0.003), whilst trained novices’ sensitivity did not significantly differ pre-to-post-domain-specific training (pre: *M* = 0.69, SD = 0.69, post: *M* = 0.60, SD = 0.88; *t*_(204)_ = 1.08, *p* = 0.527).

The interaction between time (pre-training and post-domain-general training) and group was not significant (*b* = − 0.19, *t*_(204)_ = 1.66, *p* = 0.099, 95% CI[− 0.41, 0.03]), nor were the main effects of time (post-domain-general training: *b* = 0.08, *t*_(204)_ = 0.98, *p* = 0.327, 95% CI[− 0.08, 0.23]; post-domain-specific: *b* = − 0.09, *t*_(204)_ = 1.08, *p* = 0.281, 95% CI[− 0.24, 0.07]).

These results suggest fingerprint examiners significantly outperformed novices and that domain-specific training improved fingerprint examiners’ performance—but not novices. However, domain-general training did not significantly improve either examiners’ or novices’ performance.

#### Response bias

Fingerprint examiners’ (*M* = 0.21, SD = 0.25) fingerprint response bias was significantly higher than trained novices (*M* = − 0.01, SD = 0.41; *b* = 0.25, *t*_(205.39)_ = 3.12, *p* = 0.002, 95% CI[0.08, 0.33]). The main effect of time (post-domain-specific training) was also significant such that on average, bias towards conservatism significantly increased pre-to-post after receiving domain-specific training (pre: *M* = 0.08, SD = 0.33, post: *M* = 0.09, SD = 0.33; *b* = 0.09, *t*_(204)_ = 2.06, *p* = 0.040, 95% CI[0.01, 0.19]), but not after receiving domain-general training (*b* = 0.− 0.18, *t*_(204)_ = 0.38, *p* = 0.707, 95% CI[− 0.11, 0.07]).

The interaction between time (pre-training and post-domain-general training) and group was also significant (*b* = 0.16, *t*_(204)_ = 2.42, *p* = 0.016, 95% CI[0.03, 0.29]). Examiners’ bias significantly shifted more conservatively pre-to-post after receiving domain-general training (pre: *M* = 0.12, SD = 0.26, post: *M* = 0.32, SD = 0.23; *t*_(204)_ = 3.05, *p* = 0.007), whilst novices did not significantly differ pre-to-post-domain-general training (pre: *M* = − 0.03, SD = 0.36, post: *M* = − 0.05, SD = 0.45; *t*_(204)_ = 0.38, *p* = 0.925). Whilst the interaction between time (pre-training and post-domain-specific training) and group was also significant (*b* = − 0.15, *t*_(204)_ = 2.34, *p* = 0.020, 95% CI[− 0.28, − 0.03]), neither of the follow-up comparisons were significant (novices: *t*_(204)_ = 2.06, *p* = 0.100; examiners: *t*_(204)_ = 1.25, *p* = 0.426). These results suggest that examiners displayed a tendency to respond ‘non-match’ more than novices (even increasing after domain-general training), and all participants’ response bias also shifted pre-to-post-domain-specific training but not after domain-general training.

We also conducted an exploratory analysis of the total time taken to complete the experiment between novices and examiners. We did so to explore whether this could be a potential explanation for the differences seen between the groups. We did not collect trial-level time data as response latencies can be unreliable and difficult to measure via online platforms like Qualtrics (Barnhoorn et al., 2015; Keller et al., 2009), and it was not the primary research question of interest in the present study. However, we did collect data on the total time taken to complete the survey. We, therefore, conducted a linear regression model to predict the time taken to complete the survey from group (novices or examiners) in a linear regression model. The time taken to complete the survey did not significantly differ between groups (*b* = 24,862, *t*_(102)_ = 1.91, *p* = 0.060).

Overall, Experiment 3 found that domain-specific statistical feature training improved fingerprint examiners’ comparison sensitivity—specifically on match trials (see Table 3 in Appendix). It is possible that fingerprint examiners’ non-match accuracy cannot be further improved by training as fingerprint examiners already perform exceptionally well on non-match trials (Thompson & Tangen, 2014). Further, fingerprint examiners’ response bias was generally more conservative than novices. This is consistent with previous research demonstrating that forensic science practitioners do typically have a more conservative response style than novices (Mannering et al., 2021; Towler et al., 2018; although note that accuracy is optimized when response bias is neutral). Domain-specific training did not shift either novices’ or examiners’ response bias, but domain-general training further increased examiners’ conservative response bias (although this did not have any corresponding shift in sensitivity).

However, training did not improve novices’ performance-which is inconsistent with the results of Experiments 1 and 2. To resolve this discrepancy, we pooled together the data from Experiments 1–3 and conducted an analysis of the data from all the experiments to examine the weight of evidence supporting the benefit of domain-specific and domain-combined training.

### Exploratory meta-analyses of experiments 1–3

Given the differences between the efficacy of training for novices in Experiment 3 and the first two experiments, we aimed to formalize the level of support for the impact of domain-specific and domain-combined training on novices’ performance. To do so, we pooled together the data from all the experiments and conducted a meta-analysis comparing the pre-to-post-training effects: 1) novices who received domain-combined training (Experiments 1 and 2; *N* = 213) and 2) novices who received domain-specific training (Experiments 2 and 3; *N* = 165). Note that we only included novices from Experiment 3 in the meta-analysis that completed the domain-specific training first.

Given these three experiments examined the same hypothesis (i.e. the impact of training on pre-to-post-fingerprint performance) and recorded standardized measures of d', we were able to observe the cumulative effect of training on each group across experiments. To do so, we conducted a Bayesian analysis with default Cauchy priors to examine the likelihood of the data under the null hypothesis (i.e. no difference in performance pre-to-post-training) compared to the alternative hypothesis (i.e. an increase in performance pre-to-post-training).

**Fig. 9:**
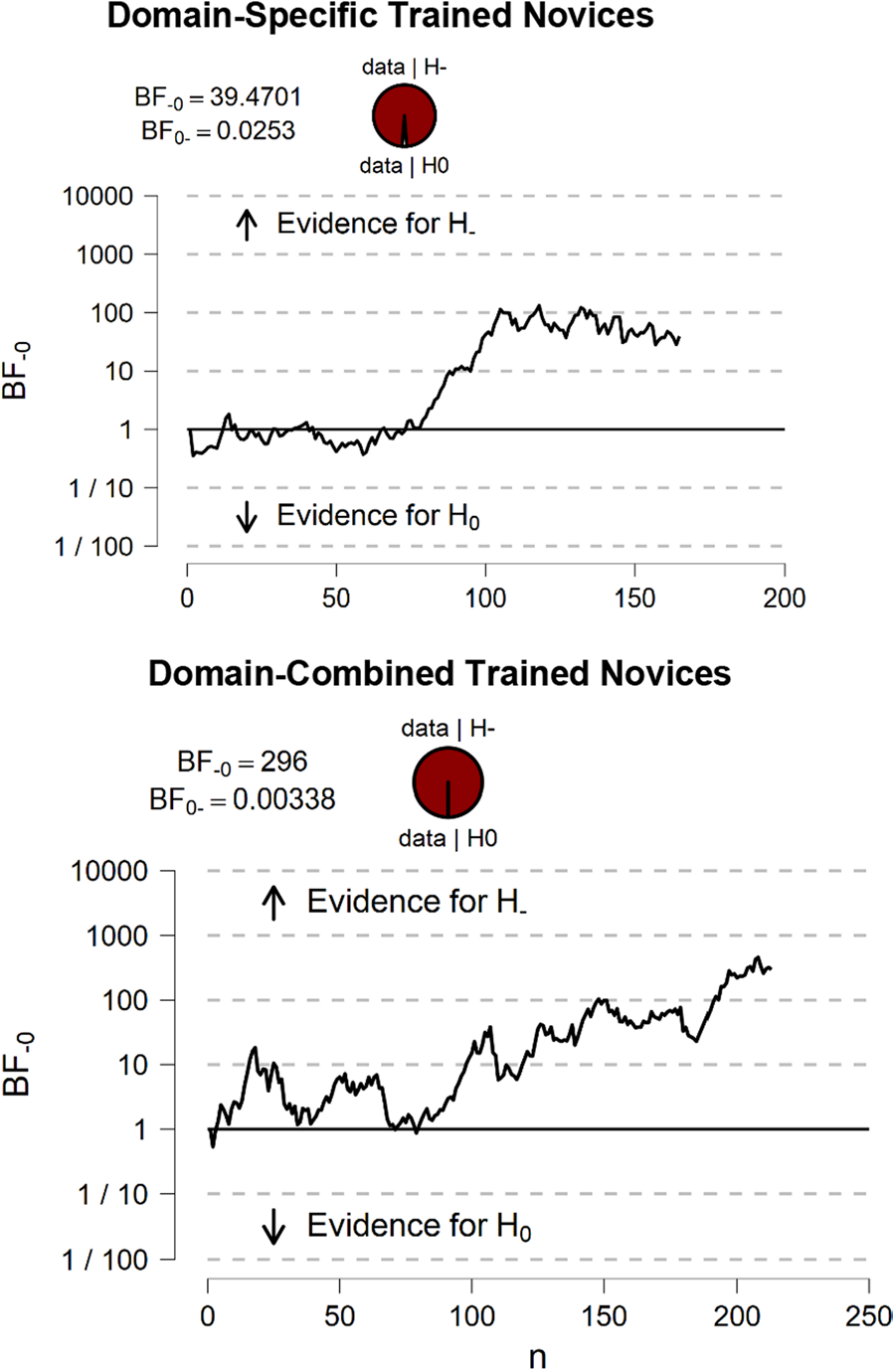
Accumulated evidence for/against the one-tailed hypothesis that fingerprint comparison sensitivity improves pre-to-post-training for novices who received domain-specific training (upper panel) or domain-combined training (lower panel)

**Fig. 10:**
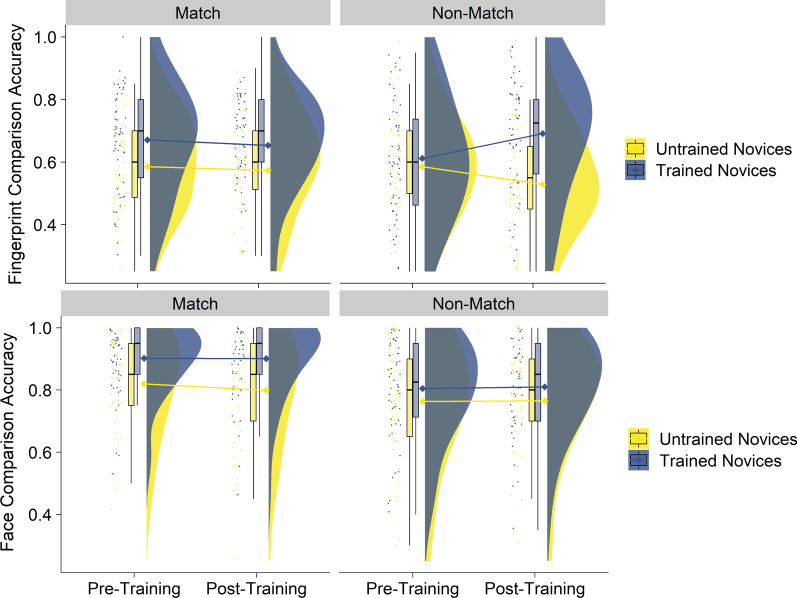
Fingerprint (upper panel) and face (lower panel) match and non-match accuracy pre-to-post-training between untrained and trained novices in Experiment 1

**Fig. 11:**
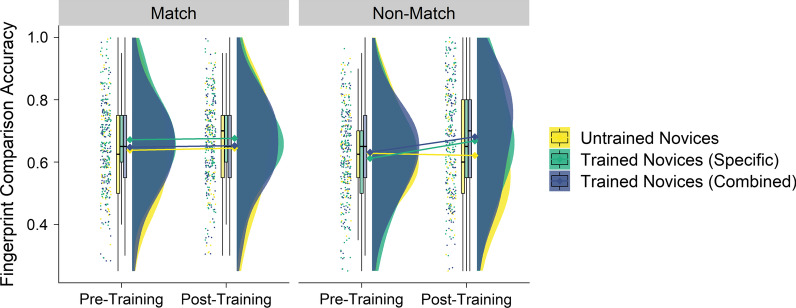
Fingerprint match and non-match accuracy pre-to-post-training between untrained novices, domain-specific trained novices, and domain-combined trained novices in Experiment 2

The cumulative support for the hypothesis that performance improved pre-to-post-domain-combined training compared to the null hypothesis, as each participant was added to the analysis, can be seen in Fig. 8. There was support in favour of the hypothesis that both domain-combined (*BF*_10_ = 296.00) and domain-specific (*BF*_10_ = 39.47) training improved novices’ performance (see Fig. 9)—providing decisive support for the former and very strong support for the latter (Wetzels et al., 2011). This indicates the data observed across Experiments 1 and 2 were 296 times more likely to occur in the case that domain-combined fingerprint comparison sensitivity improved pre-to-post-training and 40 times more likely to occur in the case that domain-specific training also improved sensitivity, than if there was no performance difference pre-to-post-training.

Overall, these results provide support for the conclusion that both domain-specific and domain-combined training improves novices’ fingerprint comparison performance. It is possible that the inconsistency in novices’ performance between Experiments 2 and 3 is spurious or due to an underpowered sample in the novice group in ‘Experiment 3’ (*N* = 52), compared to Experiment 3 (*N* = 143).

## General discussion

In this paper, we presented the results from three experiments that investigated whether statistical feature training improves the fingerprint comparison performance of novices and professional fingerprint examiners. In contrast to expert-elicited perceptual training that has previously been successful in increasing performance in visual decision-making tasks (Biederman & Shiffrar, 1987; Towler et al., 2021), we investigated the benefit of perceptual training derived from mathematical theory: training individuals to use statistically diagnostic features in visual comparison as statistically rare features provide important diagnostic information.

We found that statistical feature training improved both novice and professional performance in fingerprint comparison. The meta-analysis of the pooled data across experiments revealed that both domain-combined and domain-specific training improved fingerprint comparison performance—and both modules improved novices’ performance to a similar degree (domain-combined: 9.5% averaged over Exps. 1 and 2; and domain-specific 9.0% in Exp. 2; see Tables 1, 2 and 3 in Appendix). And whilst training improved novices’ non-match accuracy, domain-specific training resulted in a smaller but nevertheless important 4.3% increase in examiners’ match accuracy. Although examiners’ performance boost was smaller than novices, this increase could nevertheless result in avoiding important potential errors in practice (e.g. 4 out of 100 decisions). These results also suggest that domain-specific training may be sufficient to increase performance without the domain-general (i.e. face information) portion of the module.

**Fig. 12:**
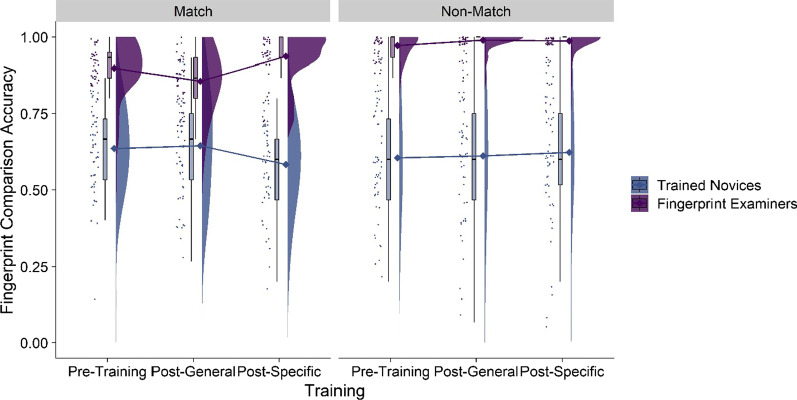
Fingerprint match and non-match accuracy pre-to-post-training between novices and fingerprint examiners who received domain-specific and domain-general training in Experiment 3

Our results also revealed that training impacted novices’ and examiners’ performance in qualitatively different ways. Whilst statistical feature training improves both novices’ and examiners’ overall sensitivity, this performance increase was driven by an increase in novices’ non-match accuracy but an increase in examiners’ match accuracy (see Tables 1, 2 and 3 in Appendix). This differential impact may not be surprising given that previous research has demonstrated that there is a limited relationship between individual performance in match and non-match trials (Megreya & Burton, 2007). It is also consistent with research demonstrating that similar training improves only novices’ non-match accuracy in face comparison (Towler et al., 2021). Statistical feature training may differentially sensitize novices and examiners to the relative similarity and dissimilarity of features that are diagnostic of same or different source exemplars. However, it is also possible that we did not observe any impact of training on examiners’ non-match accuracy due to ceiling effects as professional examiners typically already have very high non-match accuracy (Thompson & Tangen, 2014; see also Table 3 and Fig. 12 in Appendix). Nevertheless, statistical feature training does improve both novice and professional fingerprint comparison performance.

These results are also consistent with previous research demonstrating that training novices to focus on diagnostic features in visual decision-making can improve task-specific performance (Biederman & Shiffrar, 1987; Towler et al., 2021). They are further consistent with previous research demonstrating that instructing novices to focus on *statistically* diagnostic features can improve visual comparison performance (Growns & Martire, 2020a). Developing perceptual training via expert-elicitation methods requires a significant investment of time and effort. In contrast, statistically derived methods provide a new and efficient way of developing perceptual training in domains where statistical databases exist. Although such databases are only beginning to emerge in some domains (particularly in forensic science; Growns & Martire, 2020b; Growns et al., under review; Mnookin, 2008), this method of developing perceptual training provides an important and efficient avenue for future research.

Given that our perceptual training module takes only five and a half minutes to complete, this could also provide an efficient and cost-effective way to improve the professional performance of both new fingerprint trainees and existing practitioners. Further, as existing practitioners’ performance improved after training, this is also something that could be implemented in current practice to improve performance. Whilst research into the efficacy of existing forensic training is only beginning to emerge in some disciplines (e.g. facial examination Towler et al., 2019), no research has yet investigated this in fingerprint analysis. It is therefore not known whether existing training improves professional performance or the content of such training. It is possible that existing training does not include information on the relationship between statistical frequency and diagnosticity—and why our training improved professionals’ fingerprint comparison performance. Nevertheless, it provides a possible resource that could be used to improve the professional performance of fingerprint examiners—possibly by inclusion with regular ‘refresher’ training (e.g. Ludwig & Fraser, 2014; Mennell, 2006).

However, future research must replicate and further investigates the impact of such training on fingerprint comparison performance. One limitation of the present studies is that we had a restricted database of stimuli to test the efficacy of training. The magnitude of the training efficacy effect is contingent upon the stimuli used (see Towler et al., 2021 for similar results in face comparison)—and even what exemplars experience in casework. This technique is therefore most useful when fingerprints contain rare minutiae and is likely less effective in situations where they are not visible—but it is important to note that any boost in performance has the potential to reduce important real-world errors.

Our results also provide some support for the role of two distinct cognitive processes that lead to expertise in fingerprint identification (see Towler et al., 2021 for similar discussion in face identification). Previous research has largely posited that fingerprint expertise largely relies on non-analytical and holistic processing where examiners quickly and automatically make decisions (Busey & Vanderkolk, 2005; Growns & Martire, 2020b; Searston & Tangen, 2017; Thompson & Tangen, 2014). This hypothesis is largely based on research showing that examiners have higher fingerprint comparison performance in time-limited conditions (e.g. 2-s) than novices (Busey et al., 2016; Thompson & Tangen, 2014)—providing support for quick and automatic processing. However, examiners also show a *greater* advantage than novices when given more time to make decisions (Thompson & Tangen, 2014) and thus have the potential to engage analytical processing. Similar effects are also seen with other forensic science examiners (i.e. facial examiners; Towler et al., 2017, 2021). Unfortunately another limitation of the present studies is that we were unable to collect trial-level response latency data,  and we cannot directly determine whether training increased time taken to compare fingerprints pre-to-post-training (and thus opportunity to engage analytical processing). Nevertheless, the results from these studies suggest that featural, analytical processing could play an important role in fingerprint expertise. It will be important for future research to continue to investigate the relative contribution of analytical and non-analytical processing in forensic science expertise.

Overall, the studies reported here provide the first evidence for training that can improve both novices’ and professional fingerprint examiners’ comparison performance. It demonstrates that this improvement is achieved in qualitatively different ways between novices and professionals—improving novices’ non-match accuracy but examiners’ match accuracy. These results have important implications for the professional practice of fingerprint examiners and after demonstrating a benefit to existing experts and already provide a new resource to improve professional performance. They also have important theoretical implications for research investigating the cognitive mechanisms underpinning forensic science expertise and routes for how this expertise develops. Further research needs to examine whether similar statistical feature-based training modules can be derived for other forensic comparison domains (e.g. document or ballistics analysis), as well as other domains where this technique could be useful (e.g. radiology or banknote security; see van der Horst et al. (2021)).

## Data Availability

The pre-registration, data, and analysis scripts can be found at https://osf.io/jpxwe/.

## Appendix

Analyses below were logistic mixed-effects regression models to explore fingerprint comparison accuracy separately on match and non-match trials using the *lme4* and *lmerTest* packages in R, with the *emmeans* package used to explore any follow-up comparisons. We predicted raw accuracy at the trial level from the interaction between time (pre-training or post-training) and condition (relevant to each experiment), with random effects included for participant and trial which allows values to vary between participants and stimuli (Judd et al., 2012).

See Table

**Table 1:** Means, standard deviations (in brackets), and analyses on match and non-match trials pre-training and post-training between conditions in Experiment 1

	Match trial means		Non-match trial means	
Fingerprint task	Pre-training	Post-training	Pre-training	Post-training
Untrained novices	0.59 (0.49)	0.57 (0.50)	0.59 (0.49)	0.53 (0.50)
Trained novices	0.65 (0.48)	0.67 (0.47)	0.61 (0.49)	0.69 (0.40)
Face task	Pre-training	Post-training	Pre-training	Post-training
Untrained novices	0.82 (0.38)	0.80 (0.40)	0.76 (0.43)	0.77 (0.42)
Trained novices	0.90 (0.30)	0.90 (0.30)	0.81 (0.40)	0.81 (0.39)

	*b*	*t*	*p*	95% CI
*Fingerprint comparison analysis (match trials)*				
Condition	0.45	20.42	0.016	[0.09, 0.81]
Time	0.06	0.17	0.867	[− 0.62, 0.73]
Condition* time	0.03	0.19	0.854	[− 0.27, 0.32]
*Fingerprint comparison analysis (non-match trials)*				
Condition	0.88	40.61	< 0.001	[0.51, 1.26]
Time	0.34	10.07	0.284	[− 0.28, 0.97]
Condition* time	− 0.74	40.98	< 0.001	[− 1.03, − 0.45]
*Face comparison analysis (match trials)*				
Condition	0.96	30.60	< 0.001	[0.44, 1.48]
Time	0.15	0.66	0.509	[− 0.30, 0.61]
Condition* time	− 0.16	0.20	0.432	[− 0.54, 0.23]
*Face comparison analysis (non-match trials)*				
Condition	0.29	1.16	0.245	[− 0.20, 0.79]
Time	< 0.01	< 0.01	0.999	[− 0.50, 0.50]
Condition* time	− 0.03	0.160	0.873	[− 36, 0.31]

1

See Fig. ^10^

See Table

**Table 2:** Means, standard deviations (in brackets), and analyses on match and non-match trials pre-training and post-training between conditions in Experiment 2

	Match trial means		Non-match trial means	
	Pre-training	Post-training	Pre-training	Post-training
Untrained novices	0.64 (0.48)	0.64 (0.48)	0.63 (0.48)	0.62 (0.49)
Trained novices (Specific)	0.67 (0.47)	0.68 (0.47)	0.61 (0.49)	0.67 (0.49)
Trained novices (Combined)	0.64 (0.48)	0.64 (0.48)	0.63 (0.48)	0.68 (0.47)

	*b*	*t*	*p*	95% CI
*Match trial analysis*				
Condition (Specific)	0.20	1.53	0.125	[− 0.05, 0.45]
Condition (Combined)	0.05	0.41	0.684	[− 0.20, 0.30]
Time	− 0.07	0.18	0.859	[− 0.80, 0.67]
Condition (Specific) * time	− 0.03	0.22	0.824	[− 0.25, 0.20]
Condition (Combined) * time	− 0.03	0.26	0.797	[− 0.25, 0.20]
*Non-match trial analysis*				
Condition (Specific)	0.24	1.79	0.735	[− 0.02, 0.51]
Condition (Combined)	0.32	2.38	0.017	[0.06, 0.51]
Time	0.06	0.20	0.840	[− 0.56, 0.69]
Condition (Specific) * time	− 0.30	2.69	= 0.007	[− 0.54, − 0.08]
Condition (Combined) * time	− 0.32	2.91	= 0.004	[− 0.52, − 0.08]

2

See Fig. ^11^

See Table

**Table 3:** Means, standard deviations (in brackets) and analyses on match and non-match trials pre-training and post-training between groups in Experiment 3

	Match trial means			Non-match trial means		
	Pre-training	Post-specific training	Post-general training	Pre-training	Post-specific training	Post-general training
Trained novices	0.64 (0.48)	0.58 (0.49)	0.65 (0.48)	0.61 (0.49)	0.62 (0.49)	0.61 (0.49)
Fingerprint examiners	0.90 (0.30)	0.94 (0.24)	0.86 (0.24)	0.97 (0.17)	0.99 (0.11)	0.99 (0.10)

	*b*	*t*	*p*	95% CI
*Match trial analysis*				
Time (Post-specific training)	− 0.27	0.65	0.515	[− 1.07, 0.54]
Time (Post-general training)	0.09	0.21	0.834	[− 0.72, 0.89]
Group	1.97	9.63	< 0.001	[1.57, 2.38]
Time (Specific) * group	1.09	4.45	< 0.001	[0.61, 1.56]
Time (General) * group	− 0.51	2.50	0.013	[− 0.92, − 0.11]
*Non-match trial analysis*				
Time (Post-specific training)	0.08	0.27	0.788	[− 0.52, 0.69]
Time (Post-general training)	0.02	0.07	0.945	[− 0.58, 0.62]
Group	3.88	11.21	< 0.001	[3.20, 4.56]
Time (Specific) * group	0.66	1.57	0.117	[− 0.16, 1.48]
Time (General) * group	0.95	2.12	0.034	[0.07, 1.83]

3

See Fig. ^12^

## Footnotes

1 Four questions (‘Please select the “same/different” option below’) embedded during each matching task and one additional harder attention-check question designed to prevent bots from participating (‘Please enter the second word in this sentence in the textbox below’).
